# A proportional rule for setting reimbursement prices of new drugs and its mathematical consistency

**DOI:** 10.1186/s12913-020-5055-4

**Published:** 2020-03-23

**Authors:** Afschin Gandjour

**Affiliations:** Frankfurt School of Finance & Management, Adickesallee 32-34, 60322 Frankfurt am Main, Germany

**Keywords:** Cost-effectiveness, Drugs, Innovation, Pricing, I18

## Abstract

**Background:**

Value-based pricing (VBP) of new drugs has been suggested both as a way to control health expenditures and to maximize health benefits based on the available resources. The purpose of this work is to present a simple mathematical proof showing that prices of new drugs are set in a mathematically consistent way when the sum of intervention and downstream costs is proportional to the size of health benefits. Such proportional relationship underlies the efficiency-frontier method used by the German Institute for Quality and Efficiency in Health Care (IQWiG).

**Methods:**

A proof by contradiction is presented that is based upon the following three premises: 1) total costs (intervention plus downstream costs) of existing non-dominated drugs and interventions are acceptable to decision-making bodies; 2) new drugs with health benefits in-between those of the most and second most effective existing interventions are not automatically excluded from reimbursement and are acceptable if prices are sufficiently low; and 3) value is measured on a cardinal scale.

**Result:**

The proof shows that a proportional rule sets reimbursement prices of new drugs in a mathematically consistent way.

**Conclusion:**

Based on the proof and the underlying assumptions a proportional relationship between costs and health benefits ensures mathematical consistency in VBP of drugs.

## Background

In the industrialized world there has been a trend towards value-based pricing (VBP) of new, innovative medicines [1]. According to an Organization for Economic Co-operation and Development (OECD) paper [2], VBP refers to regulation of reimbursement or pricing of pharmaceuticals on the basis of their therapeutic value. VBP has been suggested both as a way to control health expenditures and to maximize health benefits based on the available resources [3]. VBP defined in a narrow sense sets prices based on a threshold incremental cost-effectiveness ratio (ICER, the ratio of additional costs to additional health benefits) [4]. In the following, we will call a VBP rule that applies an absolute cost-effectiveness threshold ‘absolute’ rule. It supports the goal of maximizing health benefits from the resources available to the health care system. Yet, application of an absolute rule has been criticized for a lack of cost control because manufacturers may increase prices up to the point where the ICER matches the threshold [5]. Thus, the absolute rule may miss its own goal. Also, application of an absolute rule requires putting an explicit dollar value on a person’s life which may be unacceptable in some countries for historical and ethical reasons. While a value-based price is, by definition, set independently of the impact on research and development (R&D), it is still relevant to analyze implications for R&D. In short, higher prices may stimulate additional research activities but may also lead to unnecessary duplication of efforts, i.e., excessive innovations. We will revert to the R&D impact in further detail in the Discussion.

In contrast, a proportional rule for VBP sets out that costs induced by a drug should (only) increase in proportion to incremental health benefits [6], thus implying a constant trade-off between costs and health benefits. A proportional rule obviates the need to put a dollar value on a person’s life; the latter is already implicitly set by the drug’s comparators. To the best of our knowledge, the only health technology assessment or pricing agency applying a proportional rule so far is Germany’s Institute for Quality and Efficiency in Health Care (Institut für Qualität und Wirtschaftlichkeit im Gesundheitswesen, IQWiG). Specifically, a proportional rule (i.e., a constant trade-off between costs and health benefits) underlies IQWiG’s “efficiency-frontier” method [6, 7], which was developed in order to provide guidance on reimbursement prices in the German social health insurance system [8, 9]. However, the proportional rule itself is more generic and does not reflect the particular requirements of IQWiG, e.g., with regard to the cost components to be incorporated or the viewpoint to be assumed.

The purpose of this paper is to present a mathematical proof supporting the principle of a proportional rule. Specifically, the proof shows that a proportional rule sets reimbursement prices of new drugs in a mathematically consistent way. As the proof also applies to IQWiG’s efficiency-frontier method, it is able to establish its validity.

In formal logic, a mathematical system is said to be “consistent if it is impossible to prove, by means of the calculus, two sequential combinations which are mutually contradictory” [10]. Therefore, the term ‘consistency’ as used in this paper does not refer to consistency with a predefined goal such as health maximization subject to limited resources. Also, note that in this paper the proportional rule is strictly used for pricing and not for resource allocation purposes. According to the latter a yes/no funding decision is made, based on prices set by manufacturers.

## Methods

### Background

According to the ‘absolute’ rule the ICER is compared to a cost-effectiveness threshold λ [11]:

1 $$ \frac{c_{\mathrm{D}}-{c}_{\mathrm{B}}}{h_{\mathrm{D}}-{h}_{\mathrm{B}}}=\frac{\Delta  c}{\Delta  h}\le \uplambda $$

where *c*_D_ − *c*_B_ denotes incremental costs of a new drug D compared to the next most effective intervention B, and *h*_D_ − *h*_B_ denotes incremental health benefit of the new therapy, i.e., the difference in health outcomes between the new drug and the next most effective intervention.

Separating the price of the new drug from its total cost yields [11]:

2 $$ \frac{\Delta  c}{\Delta  h}=\frac{p+{c}_{\mathrm{D}-\mathrm{p}}-{c}_{\mathrm{B}}}{\Delta  h}\le \uplambda $$

where *p* is the incremental price of the new drug (or drug acquisition cost) and *c*_D − p_ denotes additional costs induced by the new drug, i.e., costs of treating adverse events and drug-related services such as counseling, monitoring, and testing; savings from avoiding morbidity; and costs from avoiding premature death.

Rearranging Eq. (2) yields the maximum acceptable incremental drug price [11]:

3 $$ p\le \Delta  h\times \uplambda -\left({c}_{\mathrm{D}-\mathrm{p}}-{c}_{\mathrm{B}}\right) $$

Note that *p* refers to the price of a drug paid over lifetime. Therefore, in order to derive the unit price, total drug spending needs to be divided by the number of units provided over lifetime.

Similar to the ‘absolute’ rule, the proportional rule not only considers drug-related costs but also savings from avoided morbidity and future costs due to life extension. The sum of savings and life extension costs is defined as downstream costs. The proportional rule states that the ICER of a new drug compared to the next most effective (non-dominated^1^) intervention should not be higher than that of the next most effective intervention compared to its next most effective (non-dominated) intervention. That is, incremental costs should increase at most proportionally to incremental effects. Note that the rule does not state that drug *prices* should increase at most proportionally to incremental effects. Also, the proportional rule does not allocate resources from scratch but uses costs of currently funded interventions as a basis.

In order to apply the proportional rule a new drug needs to have two comparators. In fact, two comparators always exist (except for the rare circumstance where they are dominated by the new medicine): one is doing nothing and the other is palliative or supportive care. This also applies to preventive drugs and vaccines, as they can always be compared against i) no prevention/vaccination with palliative treatment if needed and ii) no prevention/vaccination without palliative treatment (i.e., the natural disease course). If more than two comparators exist, a new drug should be compared to the next two effective (non-dominated) interventions.

#### Proof by contradiction

In the following, we present two proofs showing that a proportional rule sets reimbursement prices of new drugs in a mathematically consistent way as defined above. Each proof is based upon the following three premises: 1) total costs (intervention-related plus downstream costs) of existing (i.e., currently reimbursed) non-dominated drugs and interventions are acceptable to decision-making bodies (i.e., total costs are taken as given, without further adjustment); 2) new drugs with health benefits in-between those of the most and second most effective existing interventions are not automatically excluded from reimbursement and are acceptable if prices are sufficiently low; and 3) value (health benefit) is measured on a cardinal scale.

## Results

### Proposition 1

For drugs with health benefits in-between those of the most and second most effective existing interventions, consistent pricing requires costs to be proportional to the size of health benefits.

### Proof

Suppose there are two existing interventions A and C where C is more effective than A. Furthermore, assume that a new drug B has health benefits that are less than those of C and above those of A. Then, health benefits of B can be formalized as a weighted average of the health benefits of A and C with *α*_h_ ∈[0,1] being the weight given to the health benefits of A and 1 – *α*_h_ being the weight given to the health benefits of C:

4 $$ {h}_{\mathrm{B}}={\alpha}_{\mathrm{h}}\times {h}_{\mathrm{A}}+\left(1-{\alpha}_{\mathrm{h}}\right)\times {h}_{\mathrm{c}} $$

In analogy, the cost of B can be formalized as a weighted average of the costs of A and C. Let *α*_c_ ∈[0,1] be the weight given to the cost of A and 1 – *α*_c_ be the weight given to the cost of C:

5 $$ {c}_{\mathrm{B}}={\alpha}_{\mathrm{c}}\times {c}_{\mathrm{A}}+\left(1-{\alpha}_{\mathrm{c}}\right)\times {c}_{\mathrm{C}} $$

According to the proportional rule, *α*_h_ = *α*_c_ holds. Now, assume that *α*_h_ < *α*_c_, reflecting an overproportional reduction in cost (i.e., a larger willingness to pay for an additional health benefit compared to a proportional rule). Furthermore, let the degree of overproportionality be expressed by the factor 1/ *α*_c_. The cost of B then becomes:

6 $$ {c}_{\mathrm{B}}=\frac{1}{\alpha_{\mathrm{c}}}\times {\alpha}_{\mathrm{c}}\times {c}_{\mathrm{A}}+\left(1-\frac{1}{\alpha_{\mathrm{c}}}\times {\alpha}_{\mathrm{c}}\right)\times {c}_{\mathrm{C}}={c}_{\mathrm{A}} $$

This shows that an overproportional discount for costs leads to a contradictory result: costs of A and B are the same despite larger health benefits of B.

Similarly, if *α*_h_ approximates 1, i.e., A and B have approximately the same health benefits, an overproportional cost reduction will lead to costs of B below those of A, thus being contradictory.

Now, assume that the discount for costs is underproportional and let the degree of underproportionality be expressed by the factor *α*_c_*/n* where *n* → ∞. We obtain:

7 $$ {c}_{\mathrm{B}}=\frac{\alpha_{\mathrm{c}}}{n}\times {\alpha}_{\mathrm{c}}\times {c}_{\mathrm{A}}+\left(1-\frac{\alpha_{\mathrm{c}}}{n}\times {\alpha}_{\mathrm{c}}\right)\times {c}_{\mathrm{C}}\approx {c}_{\mathrm{C}} $$

Hence, we obtain the contradictory result that regardless of the reduction in health benefits by B costs of B approximate those of C.

Arguing that a proportional rule should only be applied to health losses and not to health gains does not avoid contradictory results, however. This is shown by the following proof.

### Proposition 2

For drugs with larger health benefits than an existing intervention consistent pricing requires costs to be proportional to the size of health benefits.

### Proof

Assume that a new drug D has health benefits above those of C. Furthermore, assume that incremental costs of D compared to those of C are not proportional to D’s incremental health benefits compared to those of C. The ICER of D vs. C is then calculated as:

8 $$ \beta \times \frac{c_{\mathrm{C}}-{c}_{\mathrm{B}}}{h_{\mathrm{C}}-{h}_{\mathrm{B}}}=\frac{c_{\mathrm{D}}-{c}_{\mathrm{C}}}{h_{\mathrm{D}}-{h}_{\mathrm{C}}} $$

where $$ \beta =\frac{\alpha_{\mathrm{c}}}{\alpha_{\mathrm{h}}}\ne 1 $$.

Solving for *c*_D_ we obtain:

9 $$ {c}_{\mathrm{D}}=\beta \times \frac{c_{\mathrm{C}}-{c}_{\mathrm{B}}}{h_{\mathrm{C}}-{h}_{\mathrm{B}}}\times \left({h}_{\mathrm{D}}-{h}_{\mathrm{C}}\right)+{c}_{\mathrm{C}} $$

According to proposition 1, costs of C must be a weighted average of the costs of B and D:

10 $$ {c}_{\mathrm{C}}={\alpha}_{\mathrm{c}}\times {c}_{\mathrm{B}}+\left(1-{\alpha}_{\mathrm{c}}\right)\times \left(\beta \times \frac{c_{\mathrm{C}}-{c}_{\mathrm{B}}}{h_{\mathrm{C}}-{h}_{\mathrm{B}}}\times \left({h}_{\mathrm{D}}-{h}_{\mathrm{C}}\right)+{c}_{\mathrm{C}}\right) $$

Yet, in order to derive the weighted average of the costs of B and D analogous to Eq. (5), the original cost of C must be adjusted by factor

$$ \left(1-{\alpha}_{\mathrm{c}}\right)\times \left(1-\beta \right)\times \frac{c_{\mathrm{C}}-{c}_{\mathrm{B}}}{h_{\mathrm{C}}-{h}_{\mathrm{B}}}\times \left({h}_{\mathrm{D}}-{h}_{\mathrm{C}}\right) $$

This adjustment, however, is contradictory to premise 1, i.e., the cost of C is accepted in the first place.

## Discussion

This paper presents a mathematical proof in support of a proportional rule for pricing new drugs in a VBP framework. Based on the proof and its underlying assumptions a proportional relationship between costs and health benefits ensures mathematical consistency in VBP (and effectively determines the upper threshold to costs). Any upward deviation from a proportional rule leads to violation of the first premise, i.e., prices of existing non-dominated interventions are acceptable. While the proof specifically deals with average costs and health benefits, its result also holds at the level of iterations in a Monte Carlo simulation. That is, if in a single iteration health benefits of a new drug are larger than those of an existing intervention, consistent pricing requires costs to be proportional to the size of health benefits.

It is important to point out that prices are also considered acceptable when their re-evaluation is not considered cost-effective. Such a situation arises when capacity constraints in terms of producing health technology assessments exist [13, 14]. In this case, prices of existing interventions may not adequately represent their value but still are taken as they are by the technology assessment organizations. This does not preclude that manufacturers themselves lower prices of existing interventions, e.g., due to competition for market share. The resulting prices then become those that are deemed acceptable. That is, the reimbursement level of new drugs is endogenous to existing prices (the same holds for the absolute rule). Prices of existing interventions may also change as a result of new evidence on the effectiveness of existing interventions or patent expiration, thus mandating to adapt prices of new drugs over time. Yet again, these concerns also apply to the absolute rule. On a similar note, manufacturers may reduce prices of new drugs below the proportional threshold line (e.g., due to competition for market share), resulting in a less than proportional increase in total costs in relation to the health benefits. Such decrease cannot be considered unacceptable to decision-making bodies, however, as it enhances the value of the new drug.

Still, one may argue to ‘break with the past’ and reject extrapolating past costs to the future. Yet, as the proof shows it is inconsistent to do so because contradictory results will ensue if less effective drugs are also acceptable (which is the premise of our proof). Therefore, when deciding whether a given threshold ICER is consistent or not, it is not sufficient only to envision more effective drugs but also necessary to consider less effective ones (whenever the latter are permissible in a health care system).

In jurisdictions where prices of *existing* interventions may not be acceptable and sufficient capacity for evaluation exists, it is also consistent to set prices of *existing* interventions according to the proportional rule as otherwise contradictory results may be obtained. Only in the case where the price of palliative or supportive care is assessed, the proportional rule may not applicable because of lack of two comparators. In this situation we would need to allocate resources from scratch. Such an approach requires application of an absolute rule with the limitations described in the introduction.

One may attack the second premise of the proof, by disapproving adoption of drugs with less health benefits for ethical or other reasons. However, consider the situation where there are two new drugs D and E, but D - which is shortly approved after E - turns out to be less effective than E (in an indirect treatment comparison, see, e.g., [15]). In this case, D might still be funded. In order to avoid a contradiction of the sort shown in the proof, the price of D needs to be set in a way that the cost reduction of D vs. E is proportional to the reduction in health benefits.

With regard to the third premise, the requirement that value is measurable on a cardinal scale, it is also required for the absolute rule. Clearly, aggregating a multidimensional health profile into a single, one-dimensional index with cardinal properties is a non-trivial exercise. On the other hand, the commonly used metric of the quality-adjusted life year (QALY) fulfills this property at least in theory. Based on this requirement, the relationship between willingness to pay and health benefits is linear in both decision rules (as opposed to curvilinear). Along with health benefits, decision makers may want to consider additional elements of value for VBP. For example, a special consideration may be given to breakthrough or first-to-the-market drugs because they stir up hope. Also, drugs which display a small uncertainty around the size of health benefits may obtain a price premium (conversely, a larger uncertainty would lead to a price discount). In these cases, the meaning of ‘value’ of a drug needs to be redefined. But this redefinition would not invalidate the proof as the latter, strictly speaking, only requires a cardinal scale (regardless of how value is defined).

A proportional rule requires eliminating interventions which are dominated strictly or by extension. This approach is also used for the absolute rule ([12], p., 48). Including interventions that are dominated by extension instead would lower the ICER of the next more effective intervention and thus could render an otherwise cost-effective new drug not cost-effective.

The proof shown in this paper also applies to IQWiG’s efficiency-frontier method because interventions with less benefit than their comparators (overall or in particular subgroups) are not categorically excluded from reimbursement unless they are inappropriate or inefficient (cf. §92 section 1 of Social Code Book no. 5). An underlying rationale may be that a drug with on average less benefit for the whole patient population or a particular subgroup may still provide an added benefit for certain patients, e.g., those experiencing adverse events from drugs deemed superior on average. In addition, efficiency is ensured by keeping prices sufficiently below those of comparators.

In the following, we discuss important implications of the proportional rule. When downstream costs change proportional to health benefits, downstream costs will not affect drug costs and therefore can be excluded [16]. Then, the proportional rule implies that drug costs move in proportion to health benefits. This is always the case when only one clinical event is affected [16]. In case, several clinical events are affected and a new drug reduces relatively more of a costly clinical event than its comparator, savings from avoiding clinical events will be overproportional to health benefits, resulting in a higher drug price. Conversely, when a new drug affects relatively more of a less costly clinical event than its comparator, savings will be less than proportional to health benefits.

Application of the proportional rule could, ceteris paribus, slow down the growth of health expenditures considerably. Consider a traditional measure of health benefits, which is gain in life expectancy. Female life expectancy in the record-holding country has risen for 160 years at a steady pace of almost 3 months per year [17]. Assuming a persistent trend in the future [18] and keeping the relationship between growth of health expenditures and growth of life expectancy proportional, health expenditures will increase, ceteris paribus, by less than 0.3% per year. This is much lower than the real average growth in health expenditures in OECD countries since 2012 [19].

If the goal of the health care system is to keep a fixed budget (i.e., the target growth rate of health expenditures is zero), then applying the proportional rule requires reducing prices of new and existing drugs/services compared to a situation without budget constraint. In order to preserve the proportional relationship, prices of new and existing drugs/services need to be reduced in proportion. This will yield newly acceptable price levels. Note that this approach does not violate the principle of horizontal justice [20] because health care programs do not need to be abandoned. This would only occur if the proportional rule would not be used for pricing but for resource allocation purposes. Then, of course, the concern would arise that those who bear the opportunity costs of funding the new drug may not receive treatment despite having the same need as patients eligible for the new drug. In any case, when rationing is implicit, abandoned programs are no different than under an absolute rule. If the goal of the health care system is to fix the percentage of the gross domestic product (GDP) spent on health care, then applying the proportional rule requires changing prices of all health care services (including those of new drugs) in line with GDP growth, resulting in newly acceptable price levels.

According to the proportional rule, the same absolute increase in health leads to a smaller relative health gain when the next most effective intervention is fairly effective already (compared to no intervention). Conversely, when the next most effective intervention is not very effective, the relative health gain is larger. This phenomenon can also be illustrated from another angle: If the next most effective intervention is fairly effective already, its ICER and thus the threshold, ceteris paribus, are rather small, leading to a lower value of the same absolute increase in health. Given these relationships, the proportional rule accounts for disease severity [6]. Furthermore, given the positive association of disease severity and health expenditures in the German health care system [20], prices in in high-burden disease areas (e.g., metastatic cancer) tend to be higher than in low-burden disease areas. Still, larger health benefits (e.g., larger gains in life expectancy) in the latter may change the picture. Hence, there exists a tradeoff between disease severity and size of health benefits.

While the proportional rule, strictly speaking, is only applied for the purpose of VBP, one may also analyze its impact on R&D. Despite potential concerns it is not a priori evident that a proportional rule provides fewer incentives for R&D than an absolute rule. That is, while a proportional rule provides fewer rewards and incentives for manufacturers in low-cost areas, which also tend to have a low burden [21], it provides stronger incentives in high-cost high-burden disease areas (e.g., metastatic cancer). Therefore, the impact on R&D remains ambiguous and depends on how the threshold ICER of an absolute rule compares to the indication-specific ICERs under the proportional rule.

Still, if comparators are priced at generic levels, disincentives for future investment in R&D, ceteris paribus, are smaller under the absolute rule than under the proportional rule. That is, while under the absolute rule a decrease in the price of a comparator drug leads to a same-size decrease in the price of the new drug (see Eq. (3)), under the proportional rule the decrease is larger in magnitude. In any case, both rules could provide an additional reward for investment in R&D by considering the complete lifecycle of a drug including generic entry after patent expiry. This would allow charging a higher price during the period of patent protection in compensation for a later drop in price (cf. [22, 23]). Still, one may want to provide additional incentives for innovation in agreement with what are presumably the preferences of future generations [24]. To achieve dynamic efficiency, Refoios Camejo et al. [24] suggest establishing a global innovation fund. This would require extending the definition of the ‘value’ of a drug, by incorporating the health benefits of future innovations that are not on the market yet. Still, it is controversial what the optimal investment in innovation is, given that too high incentives can lead to excessive entry of firms into so-called patent races [25].

## Conclusions

This paper presents a mathematical proof in support of a proportional rule for pricing new drugs in a VBP framework. Based on the proof and its underlying assumptions a proportional relationship between costs and health benefits ensures mathematical consistency in VBP.

## Data Availability

Not applicable.

## Abbreviations

GDP: Gross domestic product
ICER: Incremental cost-effectiveness ratio
IQWiG: Institut für Qualität und Wirtschaftlichkeit im Gesundheitswesen
OECD: Organization for Economic Co-operation and Development
QALY: Quality-adjusted life year
R&D: Research and development
VBP: Value-based pricing

## Footnotes

1 An intervention is dominated if it is worse in both outcomes and costs than an alternative option; or if it has a higher ICER than an alternative option which is more effective [12].
